# Short-Term Evaluation of Guided Bone Reconstruction with Titanium Mesh Membranes and CGF Membranes in Immediate Implantation of Anterior Maxillary Tooth

**DOI:** 10.1155/2021/4754078

**Published:** 2021-11-24

**Authors:** Xianli Wang, Guoqing Wang, Xibo Zhao, Yanchuan Feng, Huijuan Liu, Fang Li

**Affiliations:** ^1^Department of Implantology, Anyang Sixth People's Hospital, Anyang 45500, China; ^2^State Key Laboratory of Military Stomatology & National Clinical Research Center for Oral Diseases & Shaanxi Key Laboratory of Oral Diseases, Department of Prosthodontics, School of Stomatology, Air Force Medical University, 710032, Xi'an, China

## Abstract

**Purpose:**

The aim of the present prospective study was to evaluate the effect of titanium mesh and concentrated growth factor (CGF) membranes in reconstructing severe labial bone defects during immediate implantation of anterior maxillary tooth.

**Methods:**

Patients with severe defects presenting on the anterior labial bone plate of maxillary were enrolled in this study. During immediate implantation, the titanium mesh was used to maintain the space of bone graft, collagen membrane, and xenograft bone that were used to guide bone regeneration (GBR). Cone beam computed tomography (CBCT) was used to measure the height and the labial bone thickness around the implant at the time of the second stage surgery, 6 months, 1 year, and 2 years after restoration. The pink esthetic score (PES) was used to evaluate the esthetic outcomes after restoration.

**Results:**

18 patients were enrolled in this study. The survival rate of implants was 100%, and no complication was observed, except for 1 case of titanium mesh exposure which did not affect osteogenesis. In the second stage of surgery, the labial bone was completely reconstructed, and the top of the implant was covered with a small amount of new bone. The thickness of the labial bone was 3.01 mm (±0.23), 2.96 mm (±0.21), 2.93 mm (±0.19), and 2.92 mm (±0.16) at the time of the second stage surgery, 6 months, 1 year, and 2 years after restoration, respectively. The height of the marginal bone around implants was above the top of implant at the time of the second stage surgery and then reduced 0.72 mm (±0.07), 0.91 mm (±0.08), and 0.90 mm (±0.07) at the time point of 6 months, 1 year, and 2 years after restoration, respectively. The changes of bone thickness and height were statistically significant within one year, but stable after one year. The PES values showed the same tendency.

**Conclusions:**

With the limitation of the present prospective study, the combination of titanium mesh and CGF membrane could provide space maintenance for bone augmentation of alveolar bone defects and improve the bone regeneration in patients with severe labial bone defect when immediate implant of anterior maxillary.

## 1. Introduction

The first study of immediate implant placement was completed by Professor Wilfried Schulte at the German University of Tubinge in 1978 [1]. After more than 40 years of basic and clinical researches, immediate implant placement has been shown to be a safe and feasible method for restoring failing teeth [2]. Immediate implant is accepted by patients and doctors because it can reduce the treatment time and restore the patients' confidence earlier.

In the implant treatment of maxillary anterior teeth, Buser believes that a fully intact facial bone wall at the extraction site is prerequisite for immediate implant placement [3]. However, the maxilla labial cortex is usually thin, dehiscence, or fenestration. Moreover, the chronic apical granuloma, the trauma during tooth extraction, etc. might often lead to severe vertical and horizontal bone defects after tooth extraction. When immediate implant placement, the presence of a facial bone defect may result in soft tissue recession which would worsen the aesthetic outcome in the anterior maxillary area, and even cause the implant failure [4].

There were many clinical methods for buccal bone defect recovery, such as guide bone regeneration (GBR), flap surgery combined with GBR and nonsubmerged healing [5], autogenous bone chips grafting [6], GBR combined with connective tissue graft, and a coronally positioned flap [7]. Bone substitute and collagen membrane are most commonly materials in GBR, While for severe anterior maxilla bone defect encountered in the immediate implantation, it is difficult for collagen membrane to maintain a suitable and stable bone regeneration space under labial muscle pressure, since the collagen membrane is easy to collapse and generate micromotion that affects blood supply [8]. However, titanium mesh shows superior mechanical properties and biological safety. It can maintain the bone regeneration space under labial muscle pressure [8–11]. Therefore, in the present study, titanium mesh was applied to stable bone regeneration space when immediate implantation with severe horizontal bone defects. The aim of the prospective study is to evaluate the effect of titanium mesh, combined with xenograft bone, collagen membrane, and concentrated growth factors (CGF), in GBR of anterior maxilla immediate implantation.

## 2. Material and Methods

### 2.1. Patient Selection

Patients presented with hopeless teeth in the anterior maxilla, asked for an immediate implant supported restoration and showing severe horizontal bone defects of anterior maxilla, were enrolled in this prospective study. They were treated with titanium mesh and immediate implantation in a period between November 2013 and November 2016 at the Implant Department of Anyang Stomatological hospital.

**Figure 1:**
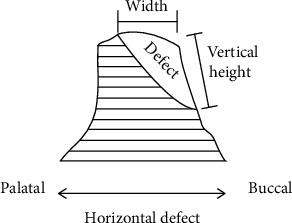
Bone defect sketch.

Inclusion criteria for the present study were no inflammation in the implant sites, insufficient width of the alveolar process, with the need for horizontal augmentation of at least 3-4 mm of buccal side, and <13 mm of vertical height in order to obtain the ideal position of the implant (Figure 1), thick gingival biotype, and good systemic and oral health and sufficient inserted torque.

Exclusion criteria were any systemic disease that could contraindicate surgery (such as uncontrolled diabetes mellitus, immunocompromised status, coagulation disorders, radiotherapy, chemotherapy, alcohol or drug abuse, and use of oral and/or intravenous aminobisphosphonates), psychiatric therapy or unrealistic expectations, heavy smokers (≥11 cigarettes/day), poor oral hygiene, pregnancy or lactation, and active periodontal infections. Further exclusion criteria for the present study were poor primary stability.

All patients had been informed about the planned treatment and had signed an informed consent form.

### 2.2. Preoperative Work-Up

Before implant placement, all patients received a session of professional oral hygiene. At the same time, cone beam computed tomography (KaVo 3D exam, Imaging Sciences International, LLC, Hatfield, Pennsylvania, USA) scans were taken to get three -dimensional (3D) evaluation of the alveolar bone process with the software of Simplant (Columbia Scientific, Inc., Columbia, MD, USA). Linear and volumetric measurements were obtained, in order to fully disclose the anatomy of the bone defect and therefore to customerize the titanium mesh for bone reconstruction. At the same time, data were imported in the software for diagnosis and implant planning. Afterwards, prosthetic-driven implants were virtually planned.

### 2.3. Surgical and Prosthetic Procedures

**Figure 2:**
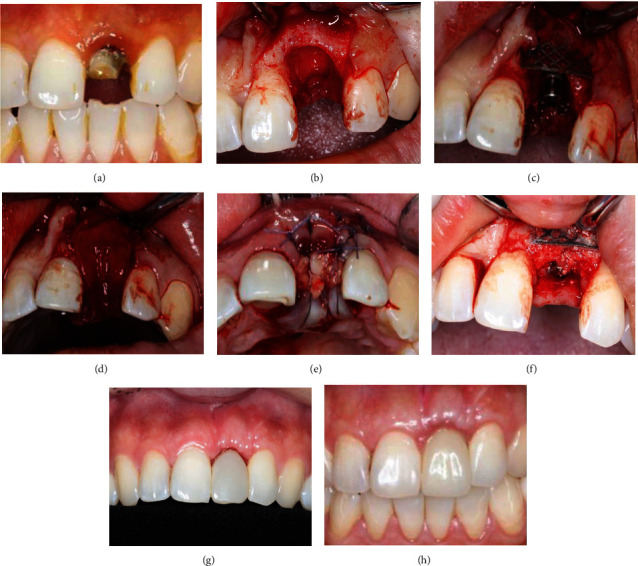
Surgical and restorative procedure of typical case 1. (a). Before tooth extraction. (b) After flap elevation and tooth extraction. (c) After implantation and titanium mesh placement. (d) Biogide placement. (e) After suture (CGF membrane covered tooth extraction fossa). (f) Taking out titanium mesh six months after implantation. (g) After temporary restoration. (h) After permanent restoration.

**Figure 3:**
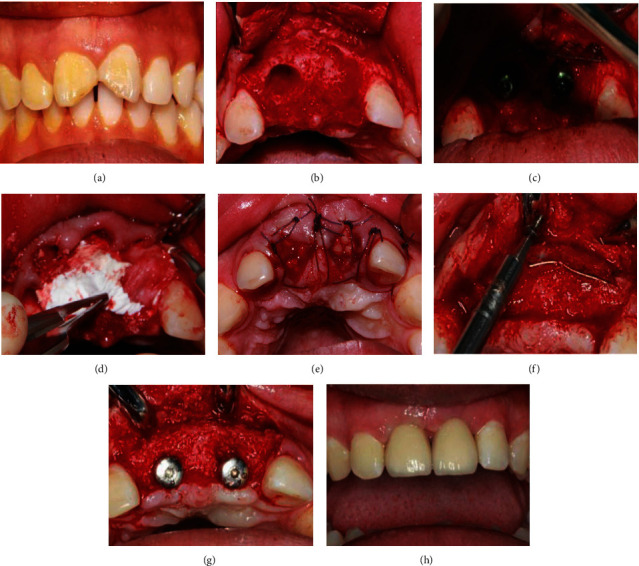
Surgical and restorative procedure of typical case 2. (a) Before teeth extraction. (b) After flap elevation and teeth extraction. (c) After implantation and titanium mesh placement. (d) Bone substitute and biogide placement. (e) After suture(CGF membrane covered tooth extraction fossa). (f) Taking out titanium mesh six months after implantation. (g) After healing abutment placement. (h) After permanent restoration.

20 ml venous blood of each patient was obtained and then centrifuged (Medifuge, Silfradent S. R. L., Santa Sofia, Italy) (2700 r/min, 10 min) to prepare CGF. After local anesthesia, disinfection, the teeth were gently extracted taking care not to further damage the remaining buccal bone wall. The alveolus was carefully cleaned in order to remove any granulation tissue. A full-thickness flap was raised to expose the defect and implant site (Figure 2(b), Figure 3(b)). Several horizontal incisions were made in the periosteum, in order to widely mobilize the flap as far as possible, in the coronal direction. After the preparation of the surgical sites using the set of helicoidal drills, the bone level implants of C-tech (C-tech implant SRL, Bologna, Italy)(Case 1) or DIO (DIO corporation, Busan, Repubulic OF Kerea)(Case 2) were placed through a computer-guided template -assisted approach in the planned position with the sufficient insertion torque, at the bone level or 1 mm deeper, according to the drilling protocol suggested by the manufacturer. Then, autogenous bone was harvested from the adjacent area, using a minimally invasive cortical bone collector (Micross, Meta, Italy). Nutricium foramina were made with a round bur to ensure vascular nutrition of the bone substitute. Autogenous bone was placed alone over the exposed implant surface. The titanium mesh (Xi'an Zhongbang titanium biology limited company, Xi′an, China) (Figures 2(c) and 3(c)) was shaped according to the size of the defects in order to maintain the space for the regenerative material. These spaces were then filled with 0.5 g particulate bone grafts (Bio–Oss, Geistlich Pharma AG, Wolhusen, Switzerland), a little autologous bone chips and CGF debris to reconstruct the width of alveolar ridge. The bone graft was over contoured to compensate final graft resorption. Then the titanium meshes were fixed with titanium screws on the bone of 2 mm outside the margin of the defect. An absorbable collagen membrane (Bio–Gide 25 × 25 *mm*, Geistlich Pharma AG, Wolhusen, Switzerland) (Figures 2(d) and 3(d)) and CGF membrane (Figures 2(e) and 3(e)) could be adapted over the titanium meshes. The soft tissues were adapted over the membranes, and care was taken in order to avoid tension during sutures. A tension-free closure was obtained (Figures 2(e) and 3(e)). Patients were prescribed oral antibiotics, amoxicillin plus clavulanic acid 0.5 g every 6 hours, for 6 days. The sutures were removed 10 days after the surgery. The titanium meshes were removed at second-stage surgery (Figures 2(f), 3(f), and 3(g)) 6 months after the first stage surgery. 1 month later, impressions were taken, and temporary resin crown restorations were provided for gingival shaping (Figure 2(g)). The temporary restorations were left for a period of 3 months, after which the definitive restorations were provided (Figures 2(h) and 3(h)). The temporary crown should be adjusted every 4 weeks, and the number of adjustment depends on the gingival shape. All definitive restorations were cemented with zinc polycarboxylate cement (Densply Sirona, Konstanz, Gemany). The patients were regularly followed up after final restoration.

### 2.4. Evaluation Criteria and Methods

#### 2.4.1. Implant Survival Rate and Complications

The implant survival rate and complications were recorded. The criteria used for successful implantation were proposed by Buser [12], as follows:

- The implant is in its original position
- There are no persistent complaints
- There is no peri-implant inflammation
- There is no implant loosening
- There is no peri-implant radiolucency

#### 2.4.2. Pink Aesthetic Score

The soft tissue outcomes were scored at the following time points: temporary restoration, permanent restoration, and 1 and 2 years after permanent restoration. The pink aesthetic score (PES) proposed by Furhauser et al. [13] was chosen as the criterion for determining the soft tissue aesthetic outcome of the implant site. The PES includes seven variables: mesial papilla, distal papilla, soft tissue level, soft tissue contour, alveolar process deficiency, soft tissue color, and texture. Using a 0-1-2 scoring system, 0 being the lowest, 2 being the highest value, the maximum achievable PES was 14. The threshold of an acceptable PES was 8. Scores of 12 or more indicated a nearly perfect outcome. All of the PES evaluations were completed by one clinician who had not participated in any related therapy process.

#### 2.4.3. Radiographic Evaluation

**Figure 4:**
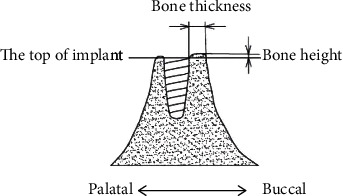
Bone thickness and bone height.

The primary outcome measures were radiographic evaluation, including CBCT and X-ray image of tooth. CBCT and X-ray were taken at the second stage of operation and 6 months, 1 year, and 2 years after permanent restoration. The thickness and height of labial bone were measured and analyzed statistically. The horizontal distance from the implant surface to the outermost edge of the buccal bone at the implant top is the bone thickness (Figure 4) [14]. The bone height is the distance from the top of the implant to the highest point of the labial bone. The highest point of the buccal bone at the second stage of operation was recorded as baseline value, and the vertical reduction from that baseline value was measured at 6 months, 1 year, and 2 years after permanent restoration, which was defined as the bone height reduction of buccal bone (Figure 4) [14].

#### 2.4.4. Statistical Analysis

Spss19.0 was used to analyze the CBCT-based data. The differences of thickness and height of buccal bone plate in different time points after operation were tested with one-way repeated measures anova. The level of significance was set at 0.05.

## 3. Results

In total, 22 patients who had been treated with titanium meshes when immediate implantation and only 18 patients (14 males, 4 females; aged between 20 and 50 years, mean age 39.5 years) were eventually enrolled in this study. Two patients were excluded because one had periodontitis, and the other had a thin gingival biotype. The survival rate of implants was 100%, and no complication was observed, except for 1 case of titanium mesh exposure which did not affect osteogenesis.

The outcome of all seven variables of the PES is shown in Table 1. From temporary restoration to permanent restoration, mesial papilla, distal papilla, soft tissue level, soft tissue contour, soft tissue color, and texture gradually approached the adjacent teeth, and the scores increased too (Table 1, Figures 2(g) and 2(h)). Within one year after the permanent restoration, slightly recession of the soft tissue level, mesial papilla, and distal papilla happened, and the root convexity slightly decreased (*P* < 0.05). However, the shape, texture, and color of soft tissue became better and better. One year later, the PES was stable (Table 1).

**Figure 5:**
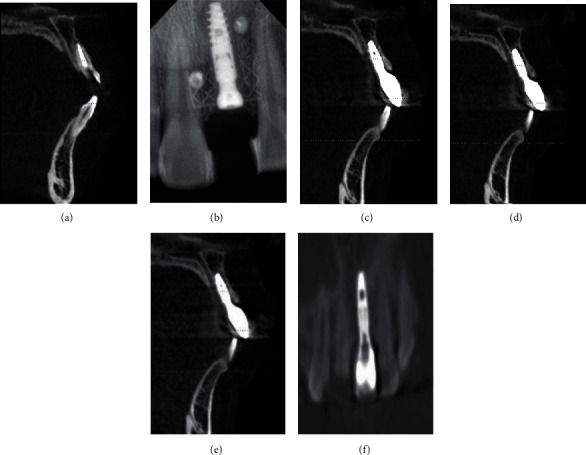
Radiographic findings. (a) Before implantation. (b) 6 months after implantation. (c) 6 months after restoration. (d) 1 year after restoration. (e) 2 years after restoration. (f) Osteogenic image around implant 2 years after restoration.

**Figure 6:**
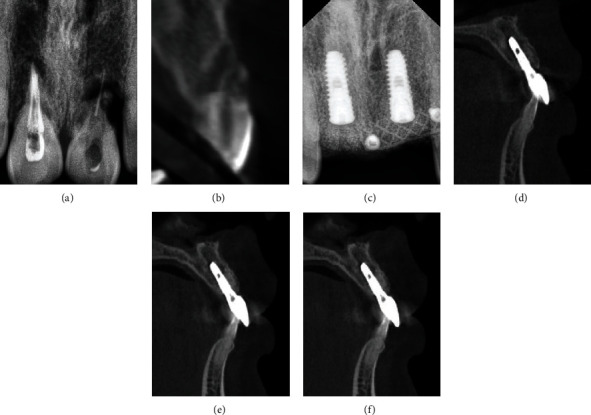
Radiographic findings. (a) X-ray before implantation. (b) CBCT before implantation. (c) 6 months after implantation. (d) Permanent restoration. (e) 1year after restoration. (e) 2 years after restoration.

The radiographic findings at the time of second stage operation and 6 months, 1 year, and 2 years after permanent restoration are presented in Figures 5(a)–5(f) and 6(a)–6(f). The labial side of the implant was exposed in the first operation, and the bone thickness was 3.01 ± 0.46 mm in the second operation. The trabeculae could be detected. The bone thickness was 3.01 mm (±0.23), 2.96 mm (±0.21), 2.93 mm (±0.19), and 2.92 mm (±0.19) at the time of the second stage surgery, 6 months, 1 year, and 2 years after restoration, respectively (Table 2). The height of the marginal bone around implants was above the top of implant at the time of the second stage surgery and then reduced to 0.72 mm (±0.07), 0.91 mm (±0.08), 0.90 mm (±0.07) at the time point of 6 months, 1 year, and 2 years after restoration, respectively (Table 2). It was found that the marginal bone resorption mainly occurred within 1 year after loading. There was no significant difference in the bone height and bone thickness of labial marginal bone between 1 year and 2 years after loading.

## 4. Discussion

When immediate implant with bone defect, GBR is the main bone augmentation method. Guided bone regeneration (GBR) is a surgical procedure that uses a graft material as a scaffold [14, 15] isolated and protected with a membrane, from the nonosteogenic cells, derived from the adjacent connective tissue. Thereby, the barrier effect of the membrane should permit only the osteogenic cells, derived from the surrounding bone and vessels, to move into the bone defect allowing for bone formation through the presence of stimulating signals. However, the collagen membrane used in GBR is so soft that easy to collapse under the pressure of labial muscles and cannot maintain a stable osteogenic space or be conducive to osteogenesis [16], consequently leading to serious aesthetic complications of anterior teeth.

Titanium meshes are nonresorbable membranes and more resistant to collapse than resorbable membranes [17, 18], and their porosity can be varied to achieve tissue compatibility which is conducive to local microcirculation on both sides of the membrane [14]. The rigidity of the titanium may work like a scaffold, maintaining the space required for the bone regeneration, even in cases of a large bone defect, such as vertical bone reconstruction [19], and muscle cannot make it deform or collapse [20, 21]. In our study, titanium mesh was used to maintain the stability of osteoblast space, which was crucial for the osteoblast growth [22]. Meanwhile, CGF was utilized to promote the growth of soft and hard tissues [23–25]. So, the severe bone defect of anterior teeth was completely repaired (Figures 2(f), 3(f), and 3(g)), and the labial bone contour was plump (Figures 2(g), 2(h), 3(g), and 3(h)). One year after implantation, the thickness of labial bone was 2.93 (±0.19 mm) (Table 2), which provided strong support for local soft tissue, and ensured the pink and white aesthetics and long-term stability of the anterior implant tooth.

It is important to close the incisions when using titanium mesh in GBR, especially with the deficiency of local soft tissue during immediate implantation. In this study, great efforts were taken to avoid titanium mesh exposure, the incisions were sutured with tension-free, and CGF membranes were covered tooth extraction fossa (Figures 2(e) and 3(e)) and placed between collagen membrane, etc. Therefore, the postoperative healing was overall excellent with only one site developing a soft tissue dehiscence with subsequent mesh exposure. However, this exposure did not affect osteogenesis, and the severe bone defect of anterior teeth was completely repaired at the second surgery. Infection rarely occurred even when the titanium mesh exposed since bacteria is not easy to adhere to the highly smooth surface of the titanium mesh [8]. Furthermore, CGF membranes covering tooth extraction fossa (Figures 2(e) and 3(e)) contain a variety of concentrated growth factors, which made up for the lack of gingiva at the top of the implant and promoted the growth of gingival tissue. CGF membranes were placed between collagen membrane and mucosal flap to cushion the friction between titanium mesh and mucosa [26]. At the same time, the high concentration of anti-infection factors in the CGF reduced the probability of postoperative infection [27–29]. So, there were few complication of titanium mesh exposure and infection in our experiment.

Adequate bone support around implant is the fundamental prerequisite for soft tissue aesthetics [26]. Nisapakultorn et al. considered that the height of gingival papilla and the level of labial marginal gingiva were determined by the height of alveolar septum and the level of marginal bone [26, 27]. In this study, the bone thickness and bone height around the implant decreased gradually within one year and became stable one year after restoration. The height of proximal and distal gingival papilla and the level of labial marginal gingiva also decreased gradually within one year and became stable one year after restoration. These results were consistent with the conclusion of nisapakultorn et al. [26].

## 5. Conclusions

With the limitation of the present prospective study, the combination of titanium mesh and CGF membrane could provide space maintenance for bone augmentation of alveolar bone defects and improve the bone regeneration in patients with severe labial bone defect when immediate implant of anterior maxillary.

## Figures and Tables

**Table 1:** PES of 20 included implants (*x*ˉ ± *s*, score, *n* = 18).

Time	Mesial papilla	Distal papilla	Soft tissue level	Soft tissue contour	Alveolar process deficiency	Soft tissue color	Soft tissue texture
Temporary restoration Permanent restoration 1 year after restoration 2 years after restoration	0.56 ± 0.12^a^ 1.87 ± 0.10^b^ 1.76 ± 0.09^c^ 1.74 ± 0.09^d^	0.59 ± 0.04^a^ 1.92 ± 0.06^b^ 1.85 ± 0.12^c^ 1.84 ± 0.12^d^	1.02 ± 0.08^a^ 1.96 ± 0.03^b^ 1.81 ± 0.05^c^ 1.80 ± 0.06^c^	0.55 ± 0.04^a^ 1.72 ± 0.06^b^ 1.82 ± 0.07^c^ 1.84 ± 0.08^d^	1.90 ± 0.04^a^ 1.93 ± 0.05^b^ 1.81 ± 0.07^c^ 1.80 ± 0.06^c^	1.39 ± 0.07^a^ 1.72 ± 0.09^b^ 1.86 ± 0.07^c^ 1.88 ± 0.08^d^	1.27 ± 0.05^a^ 1.77 ± 0.05^b^ 1.84 ± 0.03^c^ 1.85 ± 0.04^d^

Different superscripts in the same column indicate significant differences between groups (*P* < 0.05).

**Table 2:** The bone thickness and height of labial margin (*x*ˉ ± *s*, mm, *n* = 18).

	Second stage surgery	6 months	1 year	2 years
Bone thickness	3.01 ± 0.23^a^	2.96 ± 0.21^b^	2.93 ± 0.19^c^	2.92 ± 0.19^c^
Bone height reduction	Higher than implant top (baseline value)	0.72 ± 0.07^b^	0.91 ± 0.08^c^	0.90 ± 0.07^c^

Different superscripts indicate significant differences between groups (*P* < 0.05).

## Data Availability

The data used to support the findings of this study are restricted by Ethics Committee of Anyang Sixth People's Hospital in order to protect patient privacy. Xianli Wang should be contacted to request the available data through the E-mail wxlangiani@yeah.net.
